# Supplementary material for: Local emergence in Amazonia of Plasmodium falciparum k13 C580Y mutants associated with in vitro artemisinin resistance
Source: eLife. 2020 May 12;9:e51015. doi: 10.7554/eLife.51015 (PMC7217694; doi:10.7554/eLife.51015)
Supplement: Supplementary file 1. [file elife-51015-supp1.docx]

**Supplementary file 1.** Resistance genotype profiles of parasites from Guyana and French Guiana.

| **Protein** | **Mutation** | **Entire sample set from Guyana**  (n=53) | | ***pfk13*mutant** (n=13) | **O141-A*** | **R086*** |
| --- | --- | --- | --- | --- | --- | --- |
|  |  | WT frequency | Mutant frequency |  |  |  |
| **PfCRT** | C72S | 0 | 1 | S | S | S |
|  | V73V | 1 | 0 | V | V | V |
|  | M74I | 1 | 0 | M | M | M |
|  | N75E | 1 | 0 | N | N | N |
|  | K76T | 0 | 1 | T | T | T |
|  | C350R | 0.58 | 0.42 | C | C | R |
| **PfDHFR** | N51I | 0 | 1 | I | I | I |
|  | C59R | 1 | 0 | C | C | C |
|  | S108N | 0 | 1 | N | N | N |
|  | I164L | 1 | 0 | I | I | I |
| **PfDHPS** | S436F | 1 | 0 | S | S | S |
|  | A437G | 0 | 1 | G | G | G |
|  | K540E | 0 | 1 | E | E | E |
|  | A581G | 0 | 1 | G | G | G |
|  | A613T/S | 1 | 0 | A | A | A |
| **PfMDR1** | N86Y | 1 | 0 | N | N | N |
|  | Y184F | 0 | 1 | F | F | F |
|  | S1034C | 0.92 | 0.08 | S | C | S |
|  | N1042D | 0 | 1 | D | D | D |
|  | D1246Y | 0 | 1 | Y | Y | Y |

*: Parasites from French Guiana used for gene editing.
