# Supplementary material for: Local emergence in Amazonia of Plasmodium falciparum k13 C580Y mutants associated with in vitro artemisinin resistance
Source: eLife. 2020 May 12;9:e51015. doi: 10.7554/eLife.51015 (PMC7217694; doi:10.7554/eLife.51015)
Supplement: Supplementary file 3. [file elife-51015-supp3.docx]

**Supplementary file 2.** Comparison of synonymous nucleotide diversity per country.

| **Country** | **Total number of coding sites analyzed** | **Pairwise synonymous nucleotide diversity (π_syn)_** |
| --- | --- | --- |
| **Guinea** | 11320143 | 1.02 x 10^-3^ |
| **Ghana** | 11329386 | 1.01 x 10^-3^ |
| **Democratic Republic of Congo** | 11320188 | 1.03 x 10^-3^ |
| **Malawi** | 11327770 | 1.03 x 10^-3^ |
| **Cambodia** | 11324839 | 7.48 x 10^-4^ |
| **Thailand** | 11332274 | 7.40 x 10^-4^ |
| **Guyana** | 10976853 | 3.45 x 10^-4^ |
