# Supplementary material for: Local emergence in Amazonia of Plasmodium falciparum k13 C580Y mutants associated with in vitro artemisinin resistance
Source: eLife. 2020 May 12;9:e51015. doi: 10.7554/eLife.51015 (PMC7217694; doi:10.7554/eLife.51015)
Supplement: Supplementary file 4. [file elife-51015-supp4.docx]

**Supplementary file 3.** List of the sets of forward and reverse primers and dual fluorescent-labeled FAM/HEX MGB probes used.

| **SNP** | **Forward primer** | **Reverse primer** | **Probe** |
| --- | --- | --- | --- |
| **C580** | p7251  TCGTATGAAAGCATGGGTAGAG | p7252  CCATTAGTTCCACCAATGACATAAA | p7253  FAM-5'-CATCAGCTATGTGTGTTGCT-3'-MGB-EclipseDLP |
| **580Y** | p7251  TCGTATGAAAGCATGGGTAGAG | p7252  CCATTAGTTCCACCAATGACATAAA | p7254  HEX-5'-ATCATCAGCTATGTATGTTGCT-3'-MGB-EclipseDLP |
| **R539** | p7255  CCTAGAAGAAATAATTGTGGTGTTACG | p7256  GTGCCACCTCTACCCATGCT | p7257  FAM-5'-CAAATGGTAGAATTTATTG-3'-MGB-EclipseDLP |
| **539T** | p7255  CCTAGAAGAAATAATTGTGGTGTTACG | p7256  GTGCCACCTCTACCCATGCT | p7258  HEX-5'-CAAATGGTACAATTTATTG-3'-MGB-EclipseDLP |
| **ZFN WT** | p7247  AGCTTATTTTGGAAGTGCTGTATTGAA | p7248  TGACGTAACACCACAATTATTTCTTCT | p7249  FAM-5'-TGATCGTTTAAGAGATGTATGGTAT-3'-MGB-EclipseDLP |
| **ZFN bsm** | p7247  AGCTTATTTTGGAAGTGCTGTATTGAA | p7248  TGACGTAACACCACAATTATTTCTTCT | p7250  HEX-5'-TGATAGATTAAGAGACGTCTGGTAT-3'-MGB-EclipseDLP |
